# Integrating cereal genomics to support innovation in the Triticeae

**DOI:** 10.1007/s10142-012-0300-5

**Published:** 2012-11-17

**Authors:** C. Feuillet, N. Stein, L. Rossini, S. Praud, K. Mayer, A. Schulman, K. Eversole, R. Appels

**Affiliations:** 1 INRA-UBP UMR 1095 Genetics and Diversity of Cereals, Clermont-Ferrand, France; 2 Leibniz Institute of Plant Genetics and Crop Plant Research, Gatersleben, Germany; 3 Dipartimento di Produzione Vegetale, Università degli Studi di Milano, Milan, Italy; 4 Biogemma, Site de la Garenne, 90126 Chappes, France; 5 Munich Information Center for Protein Sequences/Institute for Bioinformatics and Systems Biology, Helmholtz Center Munich, Neuherberg, Germany; 6 MTT/BI Institute of Biotechnology, University of Helsinki, Helsinki, Finland; 7 Plant Genomics Laboratory, MTT Agrifood Research, Jokioinen, Finland; 8 Eversole Associates, Wyoming Road, 5207 Bethesda, USA; 9 Center for Comparative Genomics, Murdoch University, Perth, WA 6150 Australia

**Keywords:** Genetics, Genomics, Map-based cloning, Molecular breeding, Comparative genomics, Bioinformatics, Physical mapping, Wheat, Barley, Triticeae

## Abstract

The genomic resources of small grain cereals that include some of the most important crop species such as wheat, barley, and rye are attaining a level of completion that now is contributing to new structural and functional studies as well as refining molecular marker development and mapping strategies for increasing the efficiency of breeding processes. The integration of new efforts to obtain reference sequences in bread wheat and barley, in particular, is accelerating the acquisition and interpretation of genome-level analyses in both of these major crops.

## Introduction

For many years, the size and complexity of the Triticeae genomes, namely, wheat (17Gb, hexaploid), barley (5Gb, diploid), and rye (8Gb, diploid), have hampered the development of genomics and its application to breed crops with improved composition and characteristics designed to satisfy the needs of consumers, processors, and producers. Despite the recognition that a reference genome sequence is key to accelerating crop improvement, the Triticeae are the last major crops for which a complete genome sequence is not available (Feuillet and Eversole 2007). Thus, the establishment of genome sequence enabled technology platforms for the Triticeae has lagged behind advances in other cereal crops such as corn and rice. In the past decade, however, extensive efforts to develop whole-genome and chromosome-specific bacterial artificial chromosome (BAC) libraries (Allouis et al. 2003; Safar et al. 2004), extensive EST collections (ITEC http://avena.pw.usda.gov/genome/; Lazo et al. 2004; Zhang et al. 2004), transformation systems, wild germplasm and mutant collections, as well as DNA chips have permitted the establishment of large-scale genomics resources and research programs aimed at enabling high-quality sequencing of the Triticeae genomes. Given their complexity, in particular that of the hexaploid wheat genome, physical maps must be established as a scaffold for sequence assembly as it is simply not possible to achieve a reference genome sequence using whole genome shotgun approaches (Feuillet et al. 2011; Ariyadasa and Stein 2012). In the past 4 years, a number of initiatives such as the International Wheat Genome Sequencing Consortium (www.wheatgenome.org; Feuillet and Eversole 2007), the International Barley Sequencing Consortium (www.barleygenome.org), the UK wheat consortium (http://www.wheatbp.net/WheatBP/Documents/DOC_Research.php), and the European *TriticeaeGenome* FP7 project (www.triticeaegenome.eu) have developed a suite of genomic resources and knowledge to provide the foundation for physical mapping and sequencing the wheat and barley genomes. Before the development of these resources, map-based cloning was quite laborious and time-consuming, and consequently, only a few genes have been isolated in the Triticeae (Büschges et al. 1997; Brueggeman et al. 2002; 2008; Huang et al. 2003; Yan et al. 2003; Charles et al. 2009; Fu et al. 2009; Turner et al. 2005; Komatsuda et al. 2007; Taketa et al. 2008; Nair et al. 2010; Faris et al. 2010; Breen et al. 2010a; Bulgarelli et al. 2010; Yuo et al. 2012; for reviews see Stein and Graner 2004 and Krattinger et al. 2009). This was due in part to the challenge of walking efficiently along the chromosomes in repetitive sequence regions with the difficulty of identifying unique probes for screening BAC libraries. With more than 80 % of the sequence identified as transposable elements, the issues are acute in the Triticeae genomes, although the deployment of markers that cross the often unique boundaries between repetitive elements has reduced the problem to some degree (Flavell et al. 1998; Paux et al. 2010). With physical maps in hand, chromosome walking is no longer necessary and, assuming the genetic resolution is high enough, physical maps enable direct landing at the target site thereby enabling more efficient gene cloning.

In the absence of complete genome sequences and given the relatively high gene order conservation (collinearity) observed in the grass genomes, genomics studies in the Triticeae have utilized comparative genomics approaches with other grass genomes. To date, five genomes relevant to the Triticeae have been sequenced, namely rice, *Brachypodium*, sorghum, maize, and foxtail millet (IRGSP 2005; IBI 2010; Paterson et al. 2009; Schnable et al. 2009; Bennetzen et al. 2012; Zhang et al. 2012). Comparisons between these different genomes enable the identification of conserved gene regions that can support molecular marker design and identify candidate genes for traits that are well conserved between species. Hence, the high-quality rice genome sequence (IRGSP 2005) combined with the sequences of a number of genomes from the other grasses (*Brachypodium*, sorghum, maize) was used to develop molecular markers such as the Conserved Orthologous Set (COS) molecular markers (Bertin et al. 2005; Quraishi et al. 2009) and to accelerate discovery of wheat and barley genes (Fu et al. 2009; Breen and Bellgard 2010; Rustenholz et al. 2010; Krattinger et al. 2011; Distelfeld et al. 2012). However, genes such as very recently duplicated genes and those involved in end use quality (for example bread, pasta), rapidly evolving genes such as disease resistance genes, as well as genes involved in large regulatory networks are more species-specific, and thus, it is crucial to have access to genomic resources and a reference sequence of the target genome.

## Establishment of genome resources for physical mapping in the Triticeae

While for barley, whole genome BAC libraries have been produced (Schulte et al. 2011), the generation of BAC clones from flow-sorted chromosomes (Safar et al. 2004; Šafář et al. 2010) and chromosome arms (reviewed in Dolezel et al. 2012) has been key to reducing the complexity of the hexaploid wheat genome analyses. Typically, libraries of 30,000–90,000 BAC clones are generated from the flow sorting of approximately a million chromosomes (http://olomouc.ueb.cas.cz/dna-libraries/cereals) to give a coverage of over 10× for the predicted chromosome size (Dolezel et al. 2007). The BAC libraries are then fingerprinted using SnapShot labelling and analysis (Luo et al. 2003), using five restriction endonucleases, and BAC contigs are assembled generally using the FingerPrinted Contig (FPC) software (Soderlund et al. 2000). Although efficient guidelines and methodologies were established (Appels et al. 2010) and good results were obtained in wheat, as exemplified by the construction of the first physical map of the 1Gb wheat chromosome 3B (Paux et al. 2008), the length of the physical contigs was slightly lower than in small model genomes. Sequencing BAC contigs (Choulet et al. 2010) revealed a number of systematic errors in the FPC assemblies. In fact, due to the high level of repetitive DNA in the Triticeae genomes, very stringent criteria must be used to ensure a reliable assembly with the FPC software. This, in turn, often results in short contig lengths as well as an unreliable assembly in some difficult regions. To address these problems, Frenkel et al. (2010) developed a novel algorithm called Linear Topology Contig (LTC). The LTC algorithm reduces the rate of false connections and Q-clones by systematically exploring the topological contig structure and performing iterative clone clustering and ordering so that highly reliable and contigs longer than in FPC are recovered. The LTC detects “weak” connections in contigs obtained by FPC and through iterative steps undertakes their “repair.”

A further improvement in the reliability of physical map construction can be obtained using the power of next-generation sequencing technologies. KeyGene (www.keygene.com) recently developed a new approach called Whole Genome Profiling (WGP, van Oeveren et al. 2011) that is based on the sequencing of the ends of restriction endonuclease fragments after digestion of BAC pools. The application of WGP to a subset of the wheat chromosome 3B physical map (Philippe et al. 2012) demonstrated that this is a viable approach for a complex genome such as wheat and that it reduces the amount of chimeric and misassembled clones compared to the SNAPshot method while providing sequence tag information that can be used to support pooling strategies for sequencing. Thus, robust methodologies and protocols are now in place for assembling reliable physical maps in the Triticeae genomes.

## Construction of anchored physical maps

The full potential of physical maps in supporting map-based cloning and marker development for breeding can only be achieved when the BAC contigs are linked sufficiently to genetic and phenotypic maps. Thus, once physical maps have been assembled, it is essential that the BAC contigs are anchored at high density with molecular markers. This requires the development of thousands of markers followed by their assignation to the genetic and physical maps. In the Triticeae, large collaborative efforts have been deployed in the past 15 years to develop EST and SSR markers for genetic mapping enabling the construction of genetic maps that carry several hundreds of markers (wheat.pw.usda.gov/GG2/index.shtml). However, it is only with the advent of NGS technologies that high throughput development and genotyping of SNP markers progressed to a significant degree in wheat and barley. This has opened up new perspectives in anchoring strategies (Sorrells et al. 2011; Paux et al. 2012; Allen et al. 2011; Poland et al. 2012). The deployment of KASpar-based assays for SNPs in wheat provided the basis for a map of Avalon × Cadenza with 2,923 SNPs (Allen et al. 2011) and Savannah × Rialto with 1,412 SNPs (http//www.wheatisp.org). Recently, large transcriptome sequencing and resequencing efforts in Australia and the USA enabled the development of a 9K Infinium wheat SNP-chip (Akunov, personal communication). In barley, comprehensive sets of several thousands of SNP markers have been successfully developed and mapped in numerous populations (Close et al. 2009; Muñoz-Amatriaín et al. 2011; Comadran et al. 2012). With additional gene-based SNP datasets originating from programs in the UK (http://www.wheatisp.org; Allen et al. 2011) and from 5,000 COS-SNPs designed in the *TriticeaeGenome* project, a 90K SNP-chip is currently under design and should be available in the near future (Akhunov, personal communication). Focusing on the transcriptome enables a reduction in complexity that also can be achieved by the digestion of genomic DNA with restriction enzymes and sequencing of selected fragments. Genotyping by sequencing (GBS) technologies, developed originally in maize, have been applied to wheat and barley (Elshire et al. 2011; Poland et al. 2012) thereby providing another platform for DNA sequence-based ordering of reference points along the Triticeae genomes. With this approach, 34,396 SNPs were mapped by GBS in the Oregon Wolfe barley reference population, while 19,720 SNPs were mapped in the synthetic W97846 × Opata85 reference wheat population. Because they rely on sequence information, the SNP- and GBS-based approaches provide thousands of sequence tags that can be integrated into physical maps in silico thereby increasing the efficiency of anchoring strategies. This more direct integration of BAC sequencing and anchoring to genetic maps was carried out successfully in the rice genome sequencing program using BAC-end sequences (reviewed in Ariyadasa and Stein 2012), and it is currently being applied in wheat and barley (IBSC 2012). The in silico, sequence-based anchoring complements the well-established process of identifying known molecular markers in BAC pools using PCR (Paux et al. 2008) as well as the broad range of hybridization array technologies that are available (reviewed in Ariyadasa and Stein 2012).

Genetic mapping in the Triticeae genomes is difficult partly as a result of the very low rate of recombination observed in the centromeric and pericentromeric regions that can represent up to one half of an entire chromosome (Birchler et al. 2009; Kanisay and Dawe 2009). On wheat chromosome 3B, it was estimated that 90 % of crossing over occur in only 40 % of the chromosome, mostly in the telomeric regions, whereas 27 % of the chromosome did not show any crossing over (Saintenac et al. 2009). In the recombinogenic regions, sufficient resolution in ordering physical contigs to genetic maps using molecular markers can be obtained when utilizing mapping populations with high recombination frequencies. This can be achieved using recombinant inbred line (RIL) populations of several thousand individuals. Two populations of 2,600 and 4,000 RILs have been produced for this purpose in wheat (Renan × Chinese Spring) and barley (Barke × Morex), respectively, in the *TriticeaeGenome* project with the rationale of using parents that are the references for genome sequencing and physical mapping, i.e., Chinese Spring in wheat and Morex in barley. This complements the continued efforts of the International Triticeae Mapping Initiative to develop a reference population for genetic mapping in wheat using a cross between the synthetic wheat W7984 × Opata M85 population. A new population of 2,039 RILs has been established recently with a core set of 42 SSR markers used to genotype the lines and facilitate a community-base effort to build a detailed map (Sorrells et al. 2011). In addition, Sorrells et al. (2011) reported 215 doubled haploid lines that were genotyped with 1,446 molecular markers. Furthermore, multi-parent advanced generation intercross populations originating from multiple founder lines (typically 8 to 16) and therefore relying on a larger diversity and amount of recombination events than bi-parental populations have been established in wheat (Mackay and Powell 2007; Huang et al. 2012). These are currently being tested for anchoring physical maps in different projects.

In the regions near the centromeres or within large blocks of heterochromatin where little if any recombination occurs, it is not possible to reliably anchor and order a physical map. Targeting these regions is important, however, as it is now clear that genes are present all along the physical maps (Choulet et al. 2010; Rustenholz et al. 2011), including in BAC clones assigned to the centromeres and to retrotransposable element-rich regions of chromosomes. Genes, including the important vernalisation gene *Vrn-D4*, have been mapped to the centromere region (Yoshida et al. 2010). In another study, the characterization of a 0.8-Mb DNA segment from chromosome 3B that was composed almost entirely of transposable elements except for a small gene island of three conserved genes (Breen et al. 2010b). Thus, an alternative strategy for targeting centromeres and other repetitive sequence-rich regions of the chromosomes where recombination is low has been established following radiation hybrid mapping approaches that are widely used in animal genetics. Radiation hybrid mapping relies on assaying radiation-induced chromosomal fragments with molecular markers defined by the physical map under study (Riera-Lizarazu et al. 2010). The frequency of markers remaining together on the same chromosome fragment defines a measure of how physically close the markers are to each other. RH mapping was evaluated during the construction of the 3B physical map using a panel of 184 RH lines tested with 65 ISBP markers. A resolution of approximately 263 kb per break was observed (Paux et al. 2008). In particular, for the terminal bin of chromosome 3BL (3BL7-0.63-1.00), 35 loci corresponding to 32 loci were ordered and confirmed the physical map established using markers ordered by standard recombination-based mapping (Paux et al. 2008). More recently, the same panel was used to establish a high density RH map with 541 marker loci anchored to chromosome 3B spanning a total distance of 1871.9 cR (Kumar et al. 2012).

The observations on the existence of a core set of coding genes that is conserved among the grass genomes (Salse et al. 2009) have also provided a basis for anchoring chromosome (arm) specific DNA sequences to genetic and physical maps (Mayer et al. 2009; Berkman et al. 2011; Berkman et al. 2012). The so-called chromosome zipper approach was developed originally in barley to determine a virtual order of genes using inference of synteny information along the grass genomes (Mayer et al. 2009, 2011). Incorporation of chromosome arm-specific microarray hybridization information is providing an important cross-reference for the positioning of genes in this framework. In addition, cross-referencing to BAC clones that contain particular genes identified by DNA hybridisation helps in ordering BAC and FPC contigs. Typically 4,000–9,000 genic sequences per chromosome are found for wheat chromosomes, with some likely to represent gene fragments and pseudogenes (Choulet et al. 2010; Wicker et al. 2011). Following their identification, genes conserved between wheat, *Brachypodium*, rice, sorghum, and barley (Hernandez et al. 2012) can then be clustered into syntenic groups and, along with dense genetic marker information, used to define an estimated gene order in wheat and barley. The analysis also identifies predicted genes that may be unique to wheat and barley and thus might be significant in accounting for the specific agronomic attributes of these crops.

High throughput hybridization platforms can be used for anchoring physical maps if arrays of mapped markers are hybridized to labelled pools of BACs (Rustenholz et al. 2010; Hui et al. 2011; Ariyadasa and Stein 2012). The hybridization of DNA from complex genomes to microarrays with long oligonucleotides that assay different clusters of multiple SNPs, small deletion/insertion differences, copy number variants, and presence/absence variation in genomic DNA rather than single SNPs have also been used to assay polymorphisms for genotyping (Fu et al. 2010). In the case of chromosome 3B, pools of BACs were hybridised to a barley Agilent 15 K expression microarray and allowed 738 barley orthologous genes to be located to their respective BAC clones (Rustenholz et al. 2010). The study showed that 68 % of the genes identified in the study were syntenic between wheat chromosome 3B and barley 3H.

## Comparative genomics reveals unique features of the Triticeae genomes

Analyses of the grass genomes have pioneered the field of comparative genomics in plants. Early analyses of ribosomal DNA loci in the Triticeae (Dubcovsky and Dvorak 1995) indicated that despite a good conservation between the genomes, specific rearrangements occurred. Indications for this conclusion were already evident in BAC sequencing and comparison during map-based cloning projects. More recently, the construction of physical maps and whole chromosome analyses demonstrated that 30–40 % of the gene complements in wheat and barley do not reside in the conserved syntenic gene order space (Choulet et al. 2010; Mayer et al. 2011; Wicker et al. 2011; Berkman et al. 2012).

Rearrangements including deletions and expansions in the genomes of wheat have been frequent (Dubcovsky and Dvorak 2007; Wicker et al. 2011) and are considered to be the result of waves of retrotransposable elements’ movements assumed to have occurred in the recent evolutionary history of wheat (Charles et al. 2008; Choulet et al. 2010). In the case of the rDNA loci in the Triticeae, a further variable includes amplification events that increase the number of tandem copies of the genes at a given locus (Dubcovsky and Dvorak 1995). Additional mechanisms for rearrangements suggested by Wicker et al. (2011) include the repair of double-strand breaks in DNA which can include a DNA segment (plus or minus a gene sequence) from elsewhere in the genome. Although Wicker et al. (2011) argued that pseudogenes mostly arise from genome rearrangements, unique gene-domain fusions have been reported to be important in generating a gene that confers temperature-dependent resistance to wheat stripe rust wheat (Fu et al. 2009) and sensitivity to the tan spot pathogen (Faris et al. 2010). In both of these examples, protein kinase domains were fused with domains that could be recognized as being important in disease resistance gene networks.

The instability of the wheat genome was dramatically illustrated by Wicker et al. (2011) in an analysis of the short arm of chromosome 1D (1DS) which exists as a ditelosomic chromosome in a standard genetic stock of wheat used for chromosome sorting. Gene sequences for 1DS were assigned to two regions of *Brachypodium* chromosome 2, syntenic regions 1S and 1L, even though these *Brachypodium* regions are clearly differentiated when alignments with gene sequences from the ditelosomics 1AS, 1BS and 1AL, 1BL are carried out. In addition, the wheat ESTs mapping to the proximal region of 1DS in independent studies were also missing from the ditelo 1DS sequence dataset (Wicker et al. 2011).

In addition to providing the basis for analyzing conserved gene orders on a large scale (as described earlier), comparative genomics has been important in wheat and barley to define conserved features of genes, and several examples are now available for the identification of candidate genes for the phenotypes being studied. The characterization of the *Vrn* genes in wheat and barley (Yan et al. 2003, 2004, 2006; Yoshida et al. 2010) was facilitated by comparative analyses across wheat, barley, and rice. The importance of modifications in intron 1 for variation in expression of the *Vrn* genes was established through the analysis of allelic variants. A comparison to variants in *Lolium perenne* (Asp et al. 2011) showed that INDELS in several different locations within intron 1 could modify *Vrn1* expression. Additional examples include the identification of the bract suppression gene *Trd1*, a GATA transcription factor, on barley 1H through fine mapping and anchoring of the phenotype to a syntenic region in rice (Houston et al. 2012), the boron toxicity tolerance gene on barley 4H (Sutton et al. 2007), the *Rht-D1* gene region on 4D (Duan et al. 2012), and the NAC transcription factor on wheat 6B (Distelfeld et al. 2004, 2012). In the case of the NAC transcription factor, the functional attributes appeared to be quite different in rice and wheat (Distelfeld et al. 2012) and indicated extrapolating function based on shared DNA sequence structure may not always be possible.

## Gene isolation and new allele discovery

The unique features of the Triticeae genomes emphasize the importance of high-resolution mapping populations and physical maps developed in the target species to enable the de novo identification of genes that are unique to wheat and barley biology, as well as genes underlying QTLs. As genes and gene regions of interest are identified, targeted re-sequencing (Saintenac et al. 2011; Winfield et al. 2012) provides a valuable methodology for identifying new alleles in related varieties or wild relatives. The recent progress in high throughput marker development combined with new association genetics panels, physical maps, and survey sequences represent a breakthrough in map-based cloning in the Triticeae. In wheat, physical maps of chromosome 1A, 1B, 3A, 3B, and 3D (http://urgi.versailles.inra.fr/gb2/gbrowse/wheat_phys_pub/) as well as survey sequences of all 21 individual bread wheat chromosomes are already available on line (http://urgi.versailles.inra.fr/Species/Wheat/Sequence-Repository). In barley, a physical map, survey sequences of sorted chromosomes, several thousand of shotgun sequenced BAC clones and whole genome shotgun sequence assemblies were integrated to a physical/genetic genome scaffold providing an excellent template for accelerated map-based cloning and comparative genome-based candidate gene identification (IBSC 2012) (http://mips.helmholtz-muenchen.de/plant/triticeae/barleyDisclaimer.jsp; http://webblast.ipk-gatersleben.de/barley). As an example, in the Triticeae more than 15 gene and QTL projects are benefiting already from access to these resources (Table 1) and, since the reference sequence of chromosome 3B is underway, 343 scaffolds accounting for 29 Mb targeting 74 BAC-contigs sequences have already been provided to laboratories worldwide. Once candidate genes are identified, functional validation is needed to ascertain the function of the candidate. Both reverse and forward genetics approaches are now well established for wheat and barley. Stable (Agrobacterium, biolistic) and transient (VIGS) transformation systems are performed routinely in many laboratories worldwide, and mutagenized collections have been produced in both wheat and barley. In wheat, several mutant populations have been produced in diploid, tetraploid, and hexaploid genetics backgrounds, and projects are underway to establish TILLING by sequencing (Tsai et al. 2011). In barley, TILLING resources have been established (Caldwell et al. 2004; Talamè et al. 2008; Gottwald et al. 2009) that show a wide range in phenotype diversity for the discovery of genes contributing to the complex networks that underpin plant phenotypes. In parallel, natural allelic variation can also be explored by EcoTILLING approaches and associated to phenotypic variation (Xia et al. 2012).

**Table 1:** Examples of gene and QTL projects benefiting from the access to wheat and barley physical maps and sequences

Locus	Trait	Chromosome	Species	Organization	PI	Use of physical map/sequence information
*YrH52*	Stripe rust resistance	*1BS*	Emmer wheat	University of Haifa- Israel	T. Fahima	2 physical non-overlapping contigs identified (total of 3.7 Mb with estimated gap of 0.4 Mb)
*PV-QTL*	Fiber content (QTL)	*1BL*	Bread wheat	INRA - France	J. Salse	1 physical map contig (645 kb) anchored to co-segregating COS CG marker
*QSng.sfr*	Glume blotch resistance (QTL)	*3BS*	Bread wheat	University of Zurich—Switzerland	B. Keller	3 sequenced contigs identfied(1.58 Mb)
*QYld.idw*	Yield (QTL)	*3BS*	Durum wheat	University of Bologna—Italy	R. Tuberosa	Region <200 kb in the physical map containing 4 candidate genes
*Sr2*	Stem rust resistance	*3BS*	Bread wheat	CSIRO and Murdoch University	W. Spielmeyer	1 sequenced contig (1.26 Mb) spanning the Sr2 genetic interval
*SV2*	Leaf rust resistance	*3BS*	Bread wheat	INTA-Argentina	MJ. Dieguez	2 sequenced contigs (1, 2, and 3 Mb) identified with flanking markers used for marker development, screening of BAC library from resistant cultivar and candidate gene identification
*SSt1*	Solid stem (saw fly resistance)	*3B*	Durum wheat	University of Saskatchewan—Canada	C. Pozniak	17 sequenced contigs identified for marker development, 8 new cosegrating markers developed
*QFt-3B*	Flowering time (QTL)	*3BS*	Bread wheat	IEB-Czech republic	J. Safar	38 sequenced contigs identified for marker development
*Qcrs.cpi-3B*	Crown rot (QTL)	*3BS*	Bread wheat	CSIRO—Australia	C. Liu	53 sequence contigs identified for marker development
*Pm41*	Powdery mildew	*3BL*	Bread wheat	China Agricultural University—China	Z. Liu/Z. Wang	9 sequence contigs identified for marker development
*qDHY.3BL*	Drought tolerance (QTL)	*3BL*	Bread wheat	ACPFG-INRA	P. Langridge-C. Feuillet	9 sequence contigs identified for marker development and candidate gene identification
*Yr49*	Yellow rust resistance	*3DS*	Bread wheat	CSIRO—Australia	W. Spielmeyer	Contig at the proximal side identified, contig at distal side not identified yet, and cosegregating contig identified
*cul4*	Tillering	*3H*	Barley	University of Milan—Italy	L. Rossini	One BAC spanning the *cul4* genetic interval and candidate gene identified and sequenced

In Table 1, a summary is provided for traits and their respective genes that have been defined in wheat and barley. The table focuses on a list of genes on the group 1 and 3 chromosomes in wheat and barley. At a broader level, in addition to identifying the genes for disease resistance, grain quality traits have also been well defined at the DNA level. Examples include the low and high molecular weight glutenin subunit loci on the group 1 chromosomes important for flour processing quality, and the Ha locus on 5D defining the soft/hard attributes of the grain (Chantret et al. 2005; Charles et al. 2009).

## Application in molecular breeding

Increased density of markers along genetic and physical maps using array-based techniques for genes and insertion sequence-based polymorphism markers as well as GBS in wheat and barley enable breeders to perform accurate association mapping studies (Heffner et al. 2010; Liu et al. 2012). Association genetics has only been recently applied to crop plants, (Mackay and Powell 2007) yet it is an exciting alternative to conventional QTL mapping in bi-parental crosses. It draws on the principle that linkage disequilibrium (LD) tends to be maintained over many generations between loci which are physically linked to one another. Panels of non-related lines represent many cycles of historical recombinations compared to typical QTL mapping populations. LD will likely decay very rapidly with genetic distance if the panel used consists of very diverse lines; thus, correlations between marker and QTL will be identified only if the marker is tightly linked to the QTL (Paux et al. 2012; Miedaner and Korzun 2012). The number of markers needed to accomplish such studies therefore increases with the diversity of the panel, and a balance must be found between the resolution of the panel and the power to detect associations. Barley and wheat can now rely on the genomics tools described above to develop large quantities of molecular markers.

However, to take the full advantage of these resources, it is essential that adapted association panels are developed. In the past years, several elite panels have been developed for wheat and barley, notably for European germplasm. Le Couviour et al. (2011) described a panel of 195 French-grown elite winter wheat varieties. Reif et al. (2011) used a 455 central European winter wheat varieties panel for association mapping of QTL for grain yield, heading date and Fusarium Head Blight resistance, while Cockram et al. (2010) studied a 500 barley cultivar panel for 15 morphological traits. In the TriticeaeGenome project, breeders from France, the UK, and Germany established a panel of 376 varieties based on (1) general phenotypic levels of adaptation to field conditions, (2) phenotypic homogeneity, and (3) genotypic diversity. This panel has been used for genotyping and phenotyping adaptive traits such as yield, heading date, and plant height. It has been distributed to colleagues in India and Argentina who are interested in identifying new alleles in European winter wheat and is accessible for further collaboration. Thus, most of the material currently grown in Europe for wheat and barley is represented in one panel or another. The TriticeaeGenome panel and the one developed by Reif et al. (2011) are complementary resources to investigate traits in western and central European wheat germplasm that could be used to cross-validate QTL regions. Reif et al. (2011) identified QTL underlying grain yield on chromosomes 1B, 1D, 2A, 4A, 4D, 5A, 5B, and 7A and heading date on chromosomes 1B, 2B, 4B, 4D, 5A, 5D, and 7D. Moreover, the potential favorable alleles identified in these panels are more likely to be taken up by breeders as they will be already available in elite germplasm and more amenable for introduction into marker-assisted recurrent selection. Finally, genomic selection (GS) has been proposed as an alternative to use marker data in breeding that could correct some of the deficiencies in classical marker-assisted selection (Meuwissen 2009) and is the subject of further development in crop plants (Heffner et al. 2010). The TriticeaeGenome panel (376 individuals) represents a good example of the diversity in west European wheat, and it could be used to study GS in wheat. Indeed, the size of the TriticeaeGenome panel is sufficient to be divided into a training set and a validation set, and phenotypic data have already been gathered over 2 years in a total of seven locations. The panel itself is not linked to any commercial breeding programme, so it can be envisaged that both researchers and breeders use the resource.

## Bioinformatic tools and databases

The Triticeae community established a unique database, GrainGenes (http://wheat.pw.usda.gov/), to provide a suite of services for the Triticeae and oat communities, including databases, documents, tools, data files, announcements, curation, and community assistance. To date, GrainGenes stores 76 wheat genetic maps, more than 100,000 genetic markers, and approximately 271,000 wheat ESTs. These sequences can be searched through a BLAST server or by using queries to get additional information on genetic mapping data. GrainGenes also hosts the Triticeae Repeat databank that comprises 1,717 sequences of wheat transposable elements. Another database, HarvEST (http://harvest.ucr.edu/) that is highly frequented by users of the Triticeae community provides access to curated EST assemblies of wheat and barley as well as meta data linking to marker resources and orthologs of related grasses (Close et al. 2008). With the developments in physical mapping and sequencing activities, new databases have emerged to integrate outputs from studies on wheat and barley. For example, the INRA URGI Wheat database (http://urgi.versailles.inra.fr/Species/Wheat) provides access to the physical maps of The TriticeaeGenome chromosome 1BL, 1AS, 3B, and 3D as well as to the 3A physical map which is hosted at the WGGRC. Physical maps of cv. Chinese Spring chromosomes that are constructed under the framework of the IWGSC are being integrated regularly into the database (http://www.wheatgenome.org/Projects/IWGSC-Bread-Wheat-Projects/Physical-mapping/). A GBrowse displays the physical maps in relation with other datasets (e.g., genetic markers, reference sequences, QTLs, and SNPs). To date, the database stores 26 wheat genetic maps, 19,029 markers, 324 QTLs, 10,819 SNPs, and 544,529 ESTs. In addition, URGI hosts the IWGSC sequence repository that provides access to the survey sequence assemblies of the 21 chromosomes of Chinese Spring (http://urgi.versailles.inra.fr/Species/Wheat/Sequence-Repository). The URGI database is mirrored at the MIPS which host equivalent datasets for the barley genome (http://mips.helmholtz-muenchen.de/plant/barley/index.jsp).

In support of the international efforts to obtain reference sequences from rice (riceGAAS; http://ricegaas.dna.affrc.go.jp/) and the Triticeae, a versatile, easy-to-use online automated tool for annotation, a semi-automated annotation pipeline called TriAnnot has been developed (Leroy et al. 2012). Its modular architecture allows for the annotation and masking of transposable elements, the structural and functional annotation of protein-coding genes with an evidence-based quality indexing, and the identification of conserved non-coding sequences and molecular markers. To date, the TriAnnot pipeline is parallelized on a 712 CPU computing cluster that can run a 1-Gb sequence annotation in less than 26 h. When evaluated on rice and wheat data sets, TriAnnot systematically showed a higher level of reliability than other annotation pipelines that are not improved for wheat. As it is easily adaptable to the annotation of other plant genomes, TriAnnot should become a useful resource for the annotation of large and complex genomes in the future.

The rapid advances in wheat and barley provide the basis for the next steps in the efficient exploitation of the newly available resources for research and breeding. The international consortia discussed in this review have been successful in establishing an interface of cooperation to facilitate the exchange of genome sequence data and to increase the impact of advances on growers and breeding programs. Emerging technological advances in physical and genetic mapping, marker development and association genetics, bioinformatics and map-based cloning provide a foundation for step changes in germplasm development that would otherwise take a much longer period of time.
